# The impact of prudent financial policies on the urban–rural household health expenditure disparity: evidence from China

**DOI:** 10.3389/fpubh.2025.1580812

**Published:** 2025-05-27

**Authors:** Wenxin Zou, Zihui Dai, Xijie Li

**Affiliations:** ^1^School of Government, Sun Yat-sen University, Guangzhou, China; ^2^Zhejiang Business College, Hangzhou, China

**Keywords:** healthcare, urban–rural disparities, out of pocket expenditures, prudent financial policies, China

## Abstract

**Introduction:**

With the rapid development of China’s financial markets, the impact of proactive financial policies on household health investment has drawn significant attention, yet little is known about the effects of prudent financial policies on household health expenditures. We examine the effects of a prudent financial policy called the New Asset Management Regulation (NAMR). The policy aims to reduce systemic financial risks by breaking rigid payments and regulating shadow banking. The introduction of the policy expands research in this field.

**Methods:**

This study employs the intensity difference-in-differences (intensity DID) approach, utilizing data from the China Family Panel Studies (2012–2020), to assess the effects of the NAMR. We further construct interaction terms between household existing capital (both tangible and intangible) and the key explanatory variables to test the heterogeneity of the baseline results.

**Results:**

The results show that the regulation significantly affects rural households’ medical expenditures (with a 20.5% decrease in guaranteed investment and a 1.3% increase in out-of-pocket payments), but has no significant impact on urban households. Heterogeneity analysis shows urban households’ health investment reduces health spending by 0.067%, moderated negatively by tangible assets (property) and positively by intangible assets (business experience, social ties). For households with business experience, a 10% treatment intensity increase raises health spending by 5.4%. A simultaneous 10% treatment intensity and 1-unit transport spending increase boosts health expenditures by 5.8%, revealing how asset types shape urban–rural health spending differences. Furthermore, post-implementation, urban households’ education expenditures significantly increased, while migrant households experienced a rise in guaranteed investment and a decline in out-of-pocket payments, though not statistically significant.

**Discussion:**

This study highlights the multifaceted impact of prudent financial policies on household health expenditures across urban and rural areas, underscoring the need for policy formulation to address the distinct needs of urban, rural, and migrant populations, thereby laying a more solid institutional foundation for universal health coverage.

## 1 Introduction

The attainment of Universal Health Coverage (UHC) serves as a central pathway for advancing health equity and stands as a shared objective within global health governance. This is further underscored by the WHO’s 2010 World Health Report on Health Systems Financing, which explicitly articulates that achieving UHC necessitates a “shared responsibility” framework. Specifically, this entails synergistically integrating public financing mechanisms (e.g., taxation and social insurance) with rational household health expenditures (individual financing) to expand the coverage of health services. Such a dual-track financing architecture not only amplifies service accessibility but also mitigates disparities in healthcare utilization across socioeconomic strata, as evidenced by cross-national empirical analyses on UHC progress indicators (1).

Household spending on healthcare is a core expenditure, directly influencing the physical and mental health of family members while also shaping other household decisions. Existing studies have shown that economic factors, such as the level of national economic growth, government preferences for public health expenditure, and total household income, are key determinants of household health spending (2–5).

However, in China today, the financial market now more widely influences the economic investment decisions of hundreds of millions of households than ever before (64). The rising scale of health expenditures among Chinese households is partially driven by the diversification of household income sources. Since China’s economic reform and opening-up in 1978, the Chinese economy has experienced sustained rapid growth, accompanied by the gradual expansion of the financial market and a continuous increase in household participation in financial markets. Especially with the advancement of internet finance and digital financial technologies, more households have joined markets such as stocks, funds, and wealth management products, shifting from a single savings model to a diversified investment portfolio.

Against this backdrop, certain proactive financial policies, such as digital finance and inclusive finance policies, have significantly improved financial accessibility, facilitating household wealth accumulation and consumption upgrading (6–8). However, during this period of expansive growth, some potential instability factors emerged, such as shadow banking, which led to an increase in systemic risks within the financial market (65, 67, 69). In 2018, the China Banking and Insurance Regulatory Commission (CBIRC) and the China Securities Regulatory Commission (CSRC) introduced the “Measures for the New Administration of Financial Asset Management Business” (referred to as the NAMR) (66, 68). This policy aimed to break the rigidity of repayment, with fund wealth management products no longer guaranteeing the principal of household savings. This policy led to a reassessment of household investment strategies. For instance, existing studies have found that after the implementation of the NAMR, Chinese households have consistently reduced their holdings of risk assets such as funds and fixed-income products like wealth management insurance, and accumulated more flexible, disposable cash income, with their holdings of cash deposits steadily increasing (9). Therefore, the research questions of this study are: (1) How will the NAMR impact household healthcare expenditures?; (2) and how do the effects differ between urban and rural areas.

The governance objectives and operational pathways of Prudential Financial policies and proactive financial policies exhibit distinct differences. Compared to the extensive research on proactive financial policies, existing literature has paid limited attention to Prudential Financial policies, particularly their mechanisms influencing household health expenditures. Moreover, studies on the urban–rural disparities induced by such policies are even scarcer. Proactive financial policies aim to stimulate market participation and consumption, thereby promoting health-related expenditures. In contrast, prudential financial policies prioritize risk control and market stability, which may introduce greater uncertainty and subsequently suppress protective investments (10). As shown in Table 1.

**Table 1:** Analytical perspective systematic comparison.

Analysis perspective	Key independent variable	Developing countries sample region
Macroeconomic drivers	National economic growth	Viet Nam (11)
	Health insurance spending	Sri Lanka (12) Taiwan (13)
Proactive financial policies	Financial risk protection	Bangladesh (62)
	Financial inclusion	Ghana (63)
Household economics	Famliy income levels	China and India (14)

While prior studies have elucidated macroeconomic and household-level drivers of health expenditures–including national economic growth (11), health insurance spending (12, 13), and household income (14). They have paid insufficient attention to the role of financial market uncertainty, particularly as shaped by prudential policies like the NAMR, in influencing health investment decisions. Moreover, Studies utilizing Chinese samples remain scarce. Given the substantial scale of China’s financial market, it presents an ideal setting for examining the relationship between Prudential Financial policies and household health expenditures. Specifically, the existing literature has yet to fully explore how such uncertainty interacts with urban–rural disparities in risk absorption capacities, how it alters household behavioral responses following the breakdown of rigid payment guarantees, and how it triggers intergenerational tradeoffs in expenditure allocation. This study addresses key gaps in the literature, advancing our understanding of how financial regulation impacts health equity. It also develops a nuanced framework to analyze how financing market policies—and the uncertainty they generate—reshape household welfare strategies in developing economies.

Addressing this gap, this study focuses on the unique effects of Prudential Financial policies and their urban–rural heterogeneity, aiming to uncover how policy uncertainty shapes health investment decisions through differential risk absorption capacities across urban and rural households. This perspective bridges an important literature gap and simultaneously contributes new theoretical and empirical insights into financial policies and health equity.

The marginal contribution of this study lies in its early assessment of the spillover effects of the NAMR, a prudent financial policy. Firstly, this study expands the understanding of this policy. This research incorporates urban–rural differences, revealing the varying effects of financial policies on the health investment behaviors of different social groups, particularly the divergence in health investment trends between urban and rural households. This exploration not only enriches the perspectives on the impact of financial policies but also provides more detailed guidance for policymakers, emphasizing the need to adjust social security policies in tandem with financial reforms to promote the achievement of universal health coverage. Secondly, the study offers a new perspective and empirical evidence by focusing on the effects of financial market development and household financial investment uncertainty on the differences in urban–rural health expenditures. This study broadens the relationship between financial policies and health equity, focusing on the indirect effects of policies on health expenditures for urban and rural households.

Existing research confirms that the structural drivers of health inequities in China are fundamentally embedded within the urban–rural dual system (15). Building upon this institutional context, this study constructs an analytical framework centered on urban–rural disparities, specifically examining the heterogeneous impacts of Prudential Financial policies on household health expenditures.

Potential mechanisms can be explored from two theoretical perspectives. First, the health capital theory posits that health is a form of capital, and for households, increasing investment in personal health can effectively maintain and enhance future health and work capacity. Therefore, if households have more disposable income, they may be inclined to increase health-related expenditures, such as purchasing more health insurance (including both social medical insurance and commercial health insurance) to mitigate the risk of illness. According to this theory, the NAMR policy is expected to guide households in holding more cash income, thereby promoting private investments in health and indirectly advancing the achievement of universal health coverage goals.

On the other hand, the poverty trap theory offers a different perspective. This theory suggests that in many developing countries, households often lack sufficient income and savings capacity, which limits their ability to invest in health. Particularly when faced with an uncertain economic environment (such as flexible financial policies following the break in rigid repayment structures), these households may opt to reduce investments rather than increase them, including in the purchase of additional health insurance. In other words, household investment behavior, including health investments, may decline overall. If the poverty trap hypothesis holds, the NAMR could lead to an increase in the proportion of out-of-pocket expenses for households.

More importantly, these two theories represent two distinct pathways of policy impact. If the health capital theory holds, then financial reform policies like the NAMR will promote household investments in health, suggesting a positive relationship between more flexible macroeconomic policies and household health investments, which can contribute to achieving universal health coverage. In contrast, if the poverty trap hypothesis holds, it indicates a negative relationship between flexible financial policies and household health investments, meaning such policies could hinder stable household investment in health, thereby undermining efforts to achieve universal health coverage. This implies that during China’s future economic transition, the public sector must place greater emphasis on investments in the social security system when formulating macroeconomic policies to ensure the affordability of healthcare services for households, preventing excessively high out-of-pocket medical expenses that could exacerbate the economic burden of illness for families.

To assess the impact of prudent financial policies on household health expenditures, this study utilizes panel data from the China Family Panel Studies (CFPS) spanning 2012 to 2020. We apply an intensity Difference-in-Differences (DID) model to investigate the effect of the NAMR on health consumption behaviors among urban and rural households. In addition, we conduct event study analysis to perform parallel trend tests on the baseline results, and further implement counterfactual robustness checks from both sample and temporal perspectives. Additionally, considering the interregional interactions in different areas, we adjust the clustered standard errors at the provincial level. Finally, we control for the interaction of provincial and year fixed effects. To explore the reasons behind urban–rural differences, we investigate the mechanisms of result from the perspective of household existing capital, including both tangible and intangible capital. To further understand the spillover effects of the NAMR policy on urban households, we examine its impact on urban education expenditures. Finally, we also examined the changes in healthcare expenditure behavior of migrant households after the implementation of the NAMR.

The structure of the paper is as follows: Section 2 presents the literature review and hypotheses, Section 3 details the methodology and data sources, Section 4 discusses the regression results, and Section 5 concludes the study.

## 2 Theoretical framework and hypothesis

### 2.1 Theoretical framework

From a microeconomic perspective, macro-financial policies, including the NAMR, are believed to influence households’ financial asset allocation decisions (16). Financial inclusion policies have increased Chinese households’ participation in financial markets and improved investment returns (6–8). However, unlike asset pricing models, household financial decision-making is complex (17–20). Individual characteristics, such as social culture (21, 22), social capital (23), and financial literacy of household members (24), all influence household financial decisions.

The implementation of the NAMR, which increases both external uncertainty and internal asset liquidity for households, results in varying changes in household health expenditures. Funds that exit the financial market are often reallocated to expand other household investments, such as production and consumption (25–28). When the disposability of household assets improves, the health capital theory suggests that households are more likely to increase investments in health. Similar studies have indicated that digital financial technologies alleviate financing constraints and further increase health expenditures for Chinese households, particularly rural households (25, 29). However, when investment uncertainty rises, the poverty trap theory emphasizes that some households may prioritize savings to cope with economic uncertainty, reducing health investments. A classic example is that increased macroeconomic uncertainty can lead to a sustained decline in health consumption (30).

Among these, urban–rural differences are one of the most prominent areas of focus within the Chinese context (31–34). Due to factors such as the household registration system and lower financial literacy, rural households face lower accessibility to financial products (35, 36). Financial policies have a greater marginal impact on rural households (29). For instance, in the evaluation of digital financial inclusion policies, compared to urban households, rural households experienced a reduction in financing constraints and a decrease in asset vulnerability (8, 37). While the NAMR enhances the safety of the financial market, it also increases investment uncertainty. This has led to changes in Chinese household investment behavior, with a significant increase in holdings of low-risk, more liquid assets (such as cash, deposits, and government bonds) (9, 38). A large body of literature reveals systematic disparities between urban and rural areas in China, spanning capital, knowledge, and social network accumulation (39, 40). Therefore, this study adopts both theories to form hypotheses for the sample analysis.

**Figure 1:**
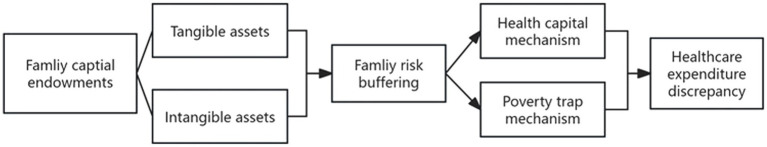
Resource endowment-risk buffering framework.

Based on resource endowment perspective, we develop a Resource Endowment-Risk Buffering (RERB) model to analyze the impacts of the New Asset Management Regulations (NAMR). Asset endowments encompass both tangible assets (e.g., per capita household income, net property value) and intangible assets (e.g., social networks, entrepreneurial experience), collectively forming a household’s risk buffer pool. Tangible assets may either directly promote household health investment or create a substitution effect on such expenditures. While intangible assets indirectly enhance health investment capacity by converting social capital into economic resources. Consequently, the direction of NAMR’s impact on household health expenditures is contingent upon variations in asset endowments. NAMR influences households’ risk buffering capacity by restructuring their asset endowment composition, thereby determining whether they can embark on health capital accumulation trajectories or fall into poverty traps due to risk aversion. At the macro level, this mechanism manifests as systematic disparities in health expenditure levels across households. The mechanism is illustrated in the Figure 1.

#### 2.1.1 Health capital mechanism

According to this model, investing in health yields long-term returns, including increased ability to work and reduced future health care costs. Under the health capital mechanism, the implementation of NAMR will not reduce the importance of family health. By breaking down rigid repayment structures, households follow health capital mechanisms to maintain or increase healthy investments, thereby adding certainty in an uncertain economic environment. This assumption applies especially to families with a certain level of income and rich social networks, especially those with a steady source of income.

#### 2.1.2 Poverty trap mechanism

This pathway suggests that poverty is a self-perpetuating state, where households are unable to accumulate sufficient resources to improve their productive capacity, making it difficult to escape poverty. Poverty is not only a phenomenon but a self-reinforcing process. Due to a lack of funds and relevant knowledge, these households typically do not invest in areas such as education, health, or skills training, which could enhance productivity. Therefore, when faced with an uncertain economic environment, low-income households are more likely to engage in risk-reducing behaviors, such as increasing savings and reducing non-essential investments (e.g., in health and education), further limiting their opportunities for improving their living conditions. Middle- and low-income households may prioritize saving to reduce potential economic risks rather than investing in their health. As a result, the implementation of the NAMR could lead to a reduction in health expenditures by Chinese households, especially among low-income households, who, due to concerns over economic uncertainty, may be more inclined to increase savings rather than invest in health.

### 2.2 Hypothesis

Asset endowments play a risk-buffering role in household health expenditures. Households with fewer assets tend to be more risk averse and may fall into the poverty trap, while households with more assets are able to assess their situation more comprehensively and make more rational decisions about health investments. Specifically, urban households in China typically hold higher proportions of tangible and intangible assets (39, 41), a ‘safe asset effect’ that enhances their risk tolerance and makes them more likely to follow the health capital mechanism (H1), that is, maintaining a healthy investment level will even increase it. In contrast, rural households have fewer tangible and intangible assets and are more susceptible to prudential policy shocks, and this lower risk buffer makes them more likely to cut back on health investments to prioritize subsistence savings, thus more likely to trigger the poverty trap mechanism (H2). Based on this, we propose the core hypothesis (H) of this study, as well as the urban hypothesis (H1) and the rural hypothesis (H2). The details are as follows:

H: NAMR affects household health expenditure and this effect is moderated by household risk tolerance leading to differences between urban and rural areas;
Urban hypothesis (H1): urban households are more likely to activate the health capital mechanism. As a result, their health investment either remains stable or increases, while out-of-pocket medical expenses correspondingly remain unchanged or decrease.
Rural hypothesis (H2): rural households are more likely to trigger the poverty trap mechanism after being hit by NAMR, which in turn reduces household health investment and increases out-of-pocket healthcare costs accordingly.

## 3 Materials and methods

### 3.1 Materials

This study use data from the China Family Panel Studies (CFPS), covering the years 2012, 2014, 2016, 2018, and 2020. The CFPS is a biennial social tracking survey designed to reflect China’s economic development and social changes through tracking a representative sample of villages, households, and family members. The survey collects data on household income and expenditures, asset status, personal health conditions, utilization of healthcare services, and medical expenditure. The baseline sample of the data covers 25 provinces (autonomous regions, and municipalities directly under the central government), ensuring a broad and representative research sample. After excluding missing values, the final usable sample consists of 19, 700 households.

### 3.2 Variables

#### 3.2.1 Dependent variables

Proportion of Protective Expenditures: Protective expenditures represent the proportion of total spending allocated to health-related protection. Y1 refers to the overall proportion of protective expenditures, Y1_1 refers to the proportion of protective expenditures for urban households, and Y1_2 refers to the proportion of protective expenditures for rural households. Protective expenditure data is derived by subtracting consumption expenditures, transfer expenditures, and housing-related mortgage payments from total expenditures. The proportion of protective expenditures measures households’ attitudes toward commercial health insurance and accident insurance. It is used to assess whether Chinese households have increased their investment in health.

#### 3.2.2 Proportion of medical expenditures in total expenditures

Y2 represents the overall proportion of medical expenditures, Y2_1 represents the proportion of medical expenditures for urban households, and Y2_2 represents the proportion of medical expenditures for rural households. The medical expenditure data is obtained through the following question: “What is the amount of direct medical expenses (in RMB per year) your household paid in the past 12 months, excluding reimbursements or expected reimbursements, but including any repayable loans for medical costs?” The proportion of out-of-pocket medical expenditures in total household spending measures the share of household health-related expenditures allocated to medical costs. This indicator reflects a household’s additional financial commitment to health, specifically highlighting the major burden of medical services expenses (such as doctor visits, medications, surgeries, and hospitalizations) that are not reimbursed. The proportion of out-of-pocket medical expenses is used to assess the impact of prudent financial policies on health expenditures by Chinese households.

#### 3.2.3 Independent variables

The independent variable is the interaction between the time dummy variable and household financial participation. The time dummy variable indicates whether the Asset Management New Regulations (NAMR) were implemented. Household financial participation is measured as the ratio of household financial assets to other assets in 2016 which is the period prior to the implementation of the NAMR. Household financial participation reflects the allocation of household assets; the higher the financial participation, the greater the household’s involvement in the financial market.

#### 3.2.4 Control variables

We control for other factors that may influence medical expenditures from four aspects: household members’ health status, household assets, social networks, and household head characteristics. First, the health status of household members directly influences medical expenditures (42, 43). Second, better household assets and social capital are likely to increase both health expenditures and investment behavior (44–47). Finally, the personal characteristics of the household head may affect both financial market participation and health investment. Female household heads are less likely to participate in financial markets, but they are more inclined to invest in health (48, 49). In Chinese society, older adult individuals place more emphasis on savings and health investments (50, 51) descriptive statistics are shown in Table 2.

**Table 2:** Variable descriptive statistics.

Variables	Meaning	Unit	Obs	Mean	Std. Dev.	Min	Max
Y1	Protective expenditure/total expenditure	-	19,700	0.030	0.071	0	0.335
Y1_1	Urban	-	7,125	0.033	0.067	0	0.300
Y1_2	Rural	-	9,405	0.028	0.076	0	0.370
Y2	Healthcare expenditure/total expenditure	-	19,700	0.099	0.138	0	0.607
Y2_1	Urban	-	7,125	0.085	0.120	0	0.529
Y2_ 2	Rural	-	9,405	0.114	0.154	0	0.664
Ratio	Financial assets/other assets (2016)	-	19,700	0.680	4.577	0	103.3
Weak	Number of unhealthy individuals in household	Persons	19,700	0.190	0.493	0	4
Medsure	Number of health insurance purchases	-	19,700	0.924	0.265	0	1
Esta	Net property	Thousand	19,700	356.8	1,124	−9,770	80,000
Fix	Fixed assets	-	19,700	3.094	4.275	0	17.73
Land	Land assets	-	19,700	5.980	4.953	0	14.96
Cash	Cash and deposits	-	19,700	6.984	4.666	0	15.43
Income	Annual income per capita	Thousand	19,700	20.32	49.89	0	4,168
Debt	Debt (Logarithm)	-	19,700	2.377	4.344	0	14.51
Business	Engaged in business	-	19,700	0.087	0.282	0	1
Y3	Education expenditure/total expenditure	-	19,700	0.066	0.113	0	0.476
Social	Social interaction expenditure	-	19,700	6.665	2.478	0	22.80
Age	Age of household head	-	19,700	51.72	13.07	18	95
Gen	Gender of household head	-	19,700	0.558	0.497	0	1
Health	Health of household head	-	19,700	2.826	1.208	1	5

Thousand indicates that the unit of this indicator is 1,000 RMB.

### 3.3 Stylized facts

**Figure 2:**
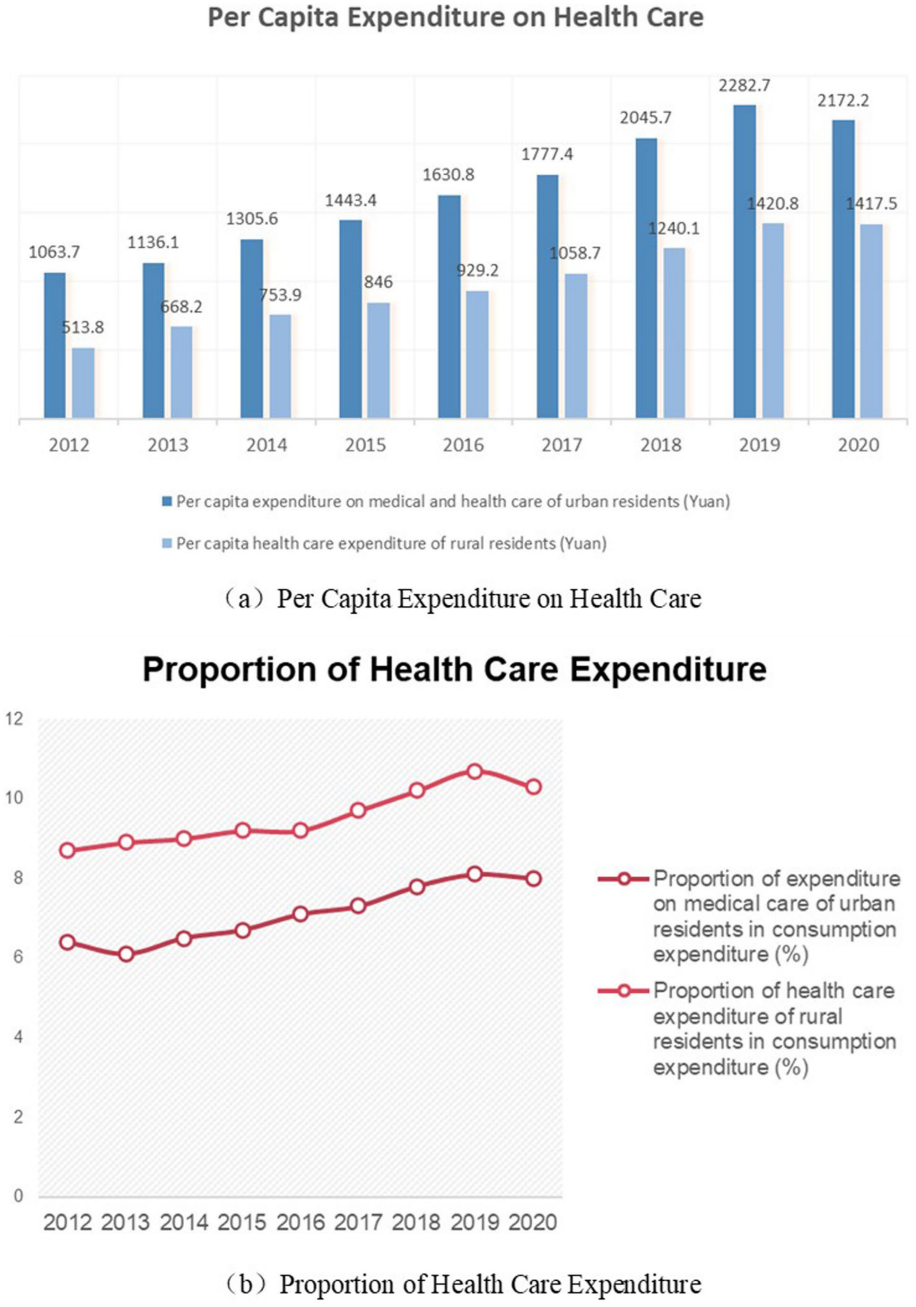
Differences in household health expenditure between urban and rural areas.

Figure 2 shows Chinese household health expenditure from 2014 to 2020.^1^ (a) Shows trends in per capita health expenditure for urban and rural households in China. (b) Shows the change in the share of total health expenditure of urban and rural households in China. Consistent with our hypothesis, there is a clear rural–urban divide in the health spending behaviors of Chinese households. Rural households spend less on health care than urban households, but health care accounts for a larger share of consumption expenditure.

**Figure 3:**
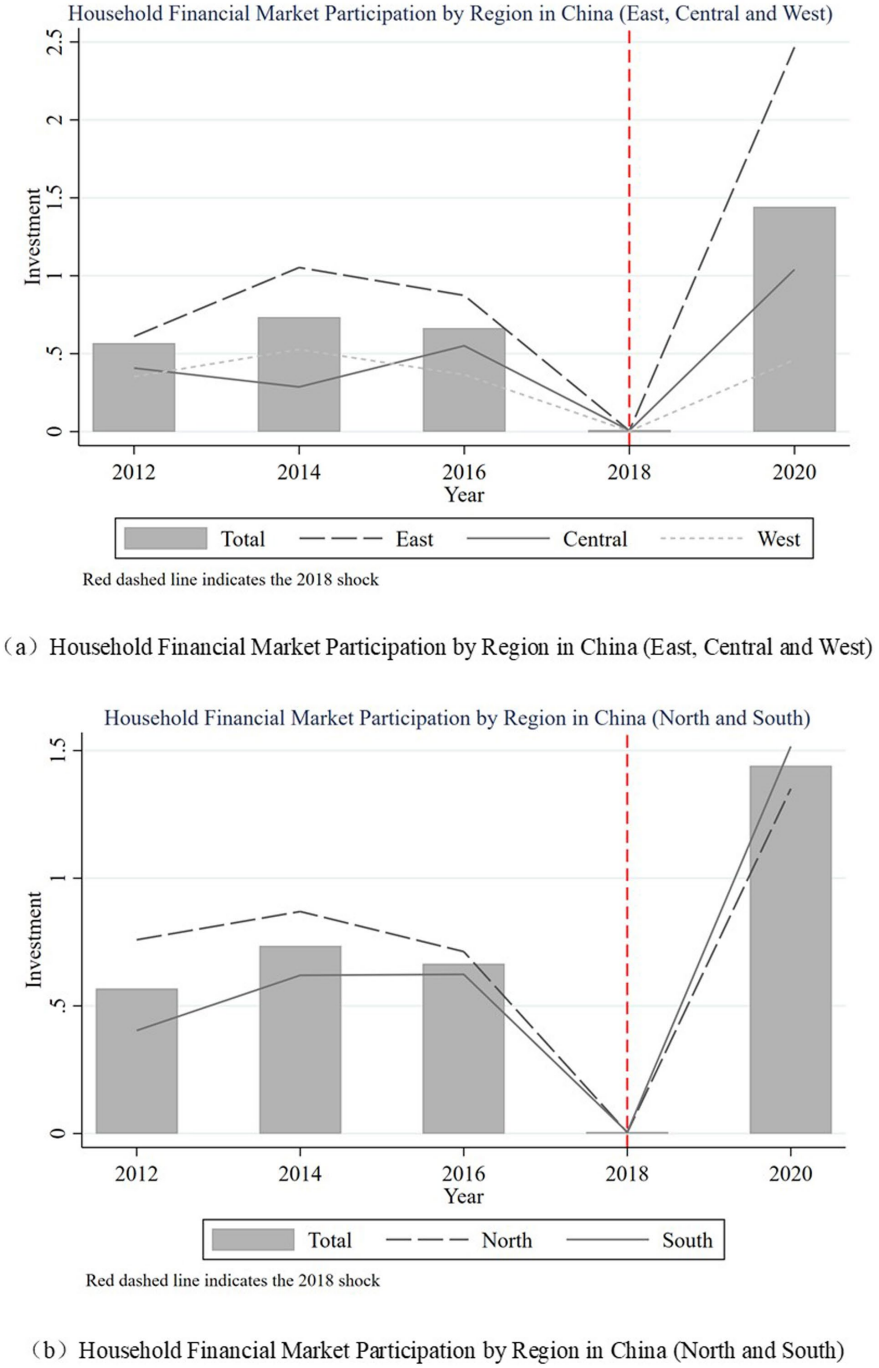
Household financial market participation in China.

Figure 3 shows the changing trend of household financial investment in China. (a) Shows the average ratio of household financial assets in eastern, central and western China. (b) Shows the average ratio of household financial assets in the south and north. As can be seen from the figure, the share of financial assets of Chinese households fell sharply during the period when the new asset regulations were announced in 2018. However, the average share of financial assets of households in the economically developed eastern regions rose to the highest level in the sample period in 2020. The average share of financial assets of families in the southern region, where clan culture is richer, will exceed that of families in the northern region in 2020. The above phenomenon suggests that physical and social capital may be important factors influencing household financial investment.

### 3.4 Empirical models

Based on NAMR, we use the intensity DID method to analyze the impact of family health expenditure. On the one hand, NAMR is a reasonable exogenous shock (52). similar to digital finance and financial inclusion policies, NAMR is issued by the central government, and its implementation is closer to an externally imposed shock than the result of internal household behavior. However, the NAMR was motivated by structural risks to the financial system and was not inherently related to household health expenditure. Finally, the policy formed a clear policy break point at the time of uniform implementation in April 2018. The NAMR policy is consistent with the sudden, global and non-targeted characteristics of exogenous shocks. On the other hand, our use of intensity DID is based on the following reality. The variance of Chinese households’ financial investment data is large, and the impact of MAMR on households with 30% financial assets is different from that on households with 10% financial assets. Traditional DID only retains binary categorical variables and can only capture average treatment effects (ATE), losing a lot of information. The intensity DID method can not only distinguish whether policies are implemented, but also quantify the intensity of policy implementation and capture the marginal treatment effect, thus better explaining and measuring the impact of policies on household health expenditure. Therefore, we referred to the study by Gao et al. (53) on NAMR, which utilized the intensity DID method to examine the impact of NAMR on health expenditure. Furthermore, since a certain number of observations of the dependent variables are zero, to ensure the statistical power of the model, this study will employ the Poisson Pseudo Maximum Likelihood (PPML) method for estimation. The model for this study is as follows:

$$Y_{nit}=\beta_{0}+\beta_{1}\times Post_{t}\times \mathrm{Participatio}n_{\mathrm{it}}+\beta_{2}\times X_{\mathrm{it}}+\gamma_{i}+\mu_{t}+\varepsilon_{\mathrm{it}}$$

$Y_{n\_mit}$ represents the dependent variable, with subscripts n and m denoting the types of the dependent variable. Specifically, n =1 refers to the proportion of protective expenditure, while n =2 refers to the proportion of out-of-pocket medical expenditure. The subscript m =1 indicates urban households, and m =2 indicates rural households. The variable Post is a time dummy variable, set to 0 for the period before the enactment of the asset management regulations and to 1 for the period after. Participation represents the degree of household financial participation, measured as the ratio of household financial assets to other assets in the pre-regulation period (2016). X represents the control variables, which include the health status of family members (measured by the number of sick individuals in the household), household assets (including net property, fixed assets, land assets, cash, and whether health insurance is purchased), per capita annual income, household debt, and household social capital (measured by the amount of money spent on social interactions, or “renqing” expenditure). In terms of household head characteristics, the model controls for the age and gender of the household head.

## 4 Result

### 4.1 Baseline results

Table 3 reports the impact of the NAMR on household protective investments. The DID coefficients from columns (1) to (4) are not significant, indicating that protective investments for both the overall sample and urban households are unaffected by the NAMR. This policy does not influence household protective behaviors overall, which may be due to substantial differences between households. The coefficient for urban households is not significant, possibly because urban households generally have more comprehensive medical coverage, and the marginal benefit of additional health insurance is lower. The findings from the subsample statistics in Table 1, where the average medical insurance holdings for urban residents are higher than for rural residents, implicitly support this possibility. Therefore, there is no change in the protective investment behavior of urban households after the implementation of the NAMR.

**Table 3:** Baseline result_1.

Variables	(1)	(2)	(3)	(4)	(5)	(6)
	Y1	Y1	Y1_1	Y1_1	Y1_2	Y1_2
Did	−0.009	−0.008	−0.010	−0.009	−0.203***	−0.205***
	(0.009)	(0.009)	(0.009)	(0.009)	(0.066)	(0.071)
Weak		0.032		0.078		0.037
		(0.045)		(0.078)		(0.071)
Medsure		0.033		0.084		−0.006
		(0.080)		(0.092)		(0.150)
Esta		0.000		0.000		0.000
		(0.000)		(0.000)		(0.000)
Fix		0.003		−0.013		0.006
		(0.007)		(0.014)		(0.010)
Land		0.019***		0.011		0.025**
		(0.006)		(0.012)		(0.010)
Cash		0.010*		−0.005		0.014
		(0.006)		(0.007)		(0.010)
Income		0.000		0.000		0.004***
		(0.000)		(0.000)		(0.001)
Debt		0.002		−0.014**		0.016**
		(0.005)		(0.007)		(0.007)
Business		0.126		0.126		0.205
		(0.098)		(0.177)		(0.164)
Social		−0.024**		−0.013		−0.038**
		(0.010)		(0.014)		(0.018)
Age		−0.002		−0.005		0.002
		(0.003)		(0.004)		(0.004)
Gen		0.024		0.108		−0.049
		(0.048)		(0.070)		(0.086)
Health		−0.013		−0.021		−0.012
		(0.020)		(0.026)		(0.035)
Y3		−0.973***		−0.731**		−1.143***
		(0.228)		(0.351)		(0.381)
Constant	−2.718***	−2.630***	−2.740***	−2.388***	−2.670***	−2.850***
	(0.005)	(0.191)	(0.006)	(0.262)	(0.008)	(0.310)
Observations	16, 660	16, 660	5, 850	5, 850	8, 020	8, 020
Control	Yes	Yes	Yes	Yes	Yes	Yes
Family FE	Yes	Yes	Yes	Yes	Yes	Yes
Year FE	Yes	Yes	Yes	Yes	Yes	Yes

Table reports the impact of NAMR on household guaranteed investment. Columns (1), (3), and (5) present the regression results for the full sample, urban sample, and rural sample, respectively. Columns (2), (4), and (6) show the regression results for the full sample, urban sample, and rural sample after including control variables. Standard errors, clustered at the county level, are reported in brackets. Significance levels are denoted as follows: *10%, **5%, and ***1%.

The DID term coefficients in columns (5) to (6) are significantly negative, suggesting that rural households, when facing investment uncertainty, reduce even their protective investments. Specifically, for rural households, for every 1 unit increase in the proportion of financial assets, protective investment decreases by 20.5% after the implementation of the NAMR.

Table 4 reports the impact of the NAMR on household out-of-pocket medical expenditures. Similar to the results in Table 3, the coefficients in columns (1) to (4) are not significant, indicating that out-of-pocket expenditure behavior for both the overall sample and urban households is unaffected by the NAMR. In line with the findings from Table 2, where rural households’ protective investments decrease, the interaction term coefficients in columns (5) to (6) are significantly positive. Specifically, for every 1 unit increase in the proportion of financial assets in rural households in 2016, medical expenditures increase by 1.3 percentage points after the implementation of the NAMR.

**Table 4:** Baseline result_2.

Variables	(1)	(2)	(3)	(4)	(5)	(6)
	Y2	Y2	Y2_1	Y2_1	Y2_2	Y2_2
Did	0.003	0.003	0.000	0.001	0.010**	0.013**
	(0.004)	(0.006)	(0.004)	(0.004)	(0.004)	(0.005)
Weak		0.117***		0.104***		0.136***
		(0.022)		(0.039)		(0.029)
Medsure		0.058		0.095*		0.066
		(0.040)		(0.054)		(0.065)
Esta		−0.000		0.000		−0.000**
		(0.000)		(0.000)		(0.000)
Fix		0.004		0.025***		−0.003
		(0.004)		(0.010)		(0.006)
Land		0.003		−0.010*		0.004
		(0.004)		(0.006)		(0.005)
Cash		−0.014***		−0.005		−0.016***
		(0.003)		(0.005)		(0.004)
Income		−0.000		0.000		−0.000*
		(0.000)		(0.000)		(0.000)
Debt		0.013***		0.013***		0.017***
		(0.003)		(0.005)		(0.004)
Business		−0.130**		−0.250*		−0.091
		(0.063)		(0.138)		(0.075)
Social		−0.010*		−0.003		−0.014*
		(0.005)		(0.008)		(0.008)
Age		0.009***		0.008***		0.008***
		(0.001)		(0.003)		(0.002)
Gen		0.025		0.025		0.041
		(0.026)		(0.050)		(0.037)
Health		−0.103***		−0.129***		−0.102***
		(0.011)		(0.019)		(0.015)
Y3		−0.970***		−0.833***		−1.127***
		(0.117)		(0.264)		(0.172)
Constant	−1.962***	−2.151***	−2.092***	−2.272***	−1.843***	−2.010***
	(0.003)	(0.107)	(0.004)	(0.195)	(0.002)	(0.147)
Observations	19, 650	19, 650	7, 105	7, 105	9, 390	9, 390
Control	Yes	Yes	Yes	Yes	Yes	Yes
Family FE	Yes	Yes	Yes	Yes	Yes	Yes
Year FE	Yes	Yes	Yes	Yes	Yes	Yes

Table reports the impact of the New Asset Management Regulations on the proportion of household medical expenditures. Columns (1), (3), and (5) present the regression results for the full sample, urban sample, and rural sample, respectively. Columns (2), (4), and (6) show the regression results for the full sample, urban sample, and rural sample after including control variables, respectively. The standard errors from clustering to the county level are enclosed in brackets. Significance levels are indicated as follows: *10%, **5%, and ***1%.

The rural household sample thus supports the H2 hypothesis. After the NAMR, rural households, when facing uncertainty, may lean towards reducing investments and increasing liquidity. This leads to an increase in out-of-pocket medical expenditures when healthcare payments are required. The differential impact of policy implementation is evident. On the one hand, rural households often face significant differences from urban households in terms of income levels, financial market participation, and social security coverage, which means the new policy may have a more direct or pronounced impact on rural households. On the other hand, the relative burden of medical expenses increases: the new regulations may make rural households more vulnerable when facing medical expenditures, or it may reflect that they are more sensitive to changes in financial assets.

### 4.2 Robustness tests

#### 4.2.1 Intensity test

**Figure 4:**
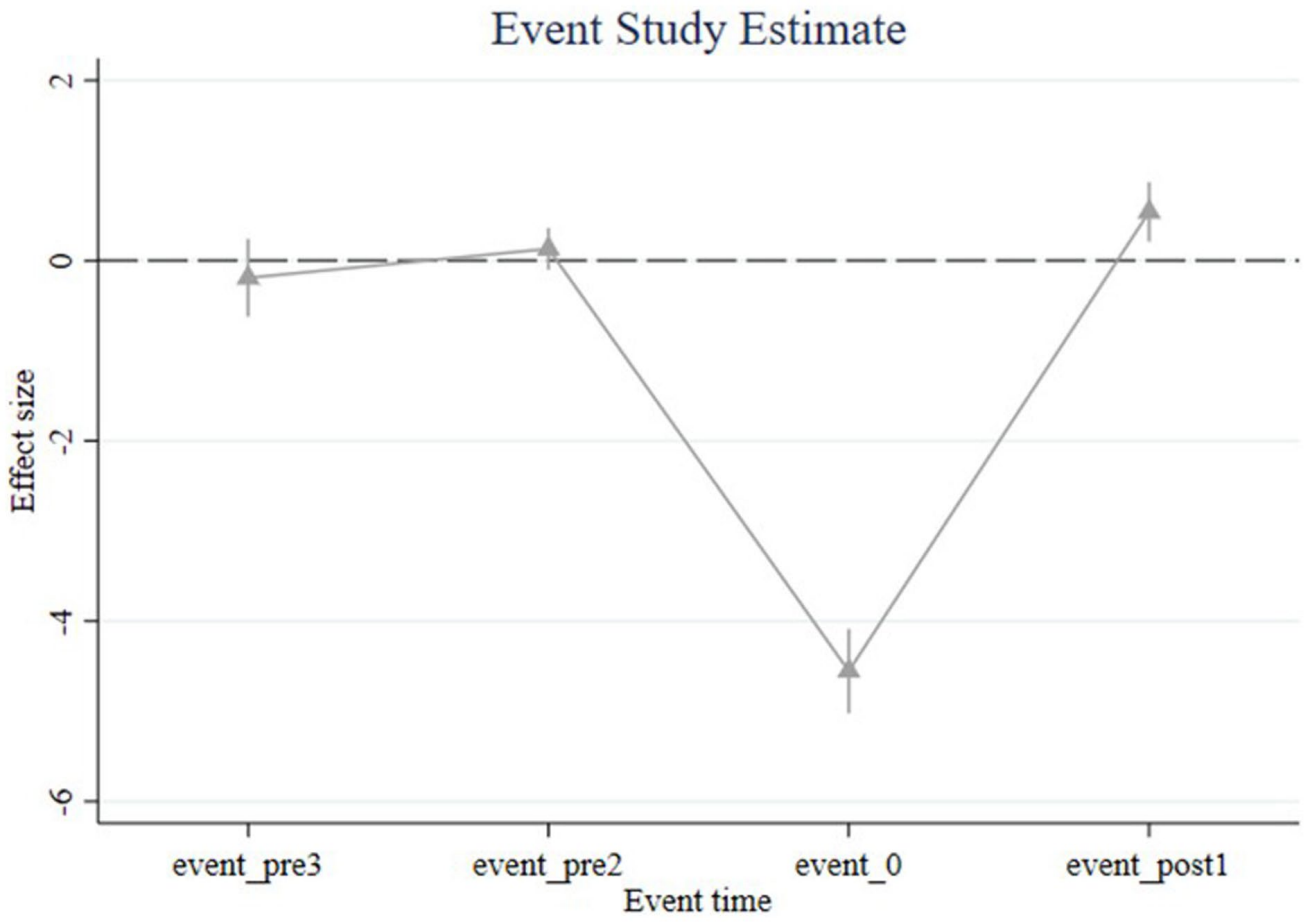
Event Study. Figure shows the results of the event analysis. The confidence interval is 5%.

The intensity variable in the intensity DID framework must capture the differential exposure to the policy shock. In our baseline specification, we proxy the intensity using the ratio of household financial assets to other assets. To validate this measure, we conduct an event-study analysis where the intensity variable itself serves as the dependent variable to evaluate NAMR’s impact on financial asset shares. As shown in Figure 4, the immediate decline in Chinese households’ financial asset ratios following NAMR implementation confirms the validity of our intensity measure.

#### 4.2.2 Parallel trend test

**Figure 5:**
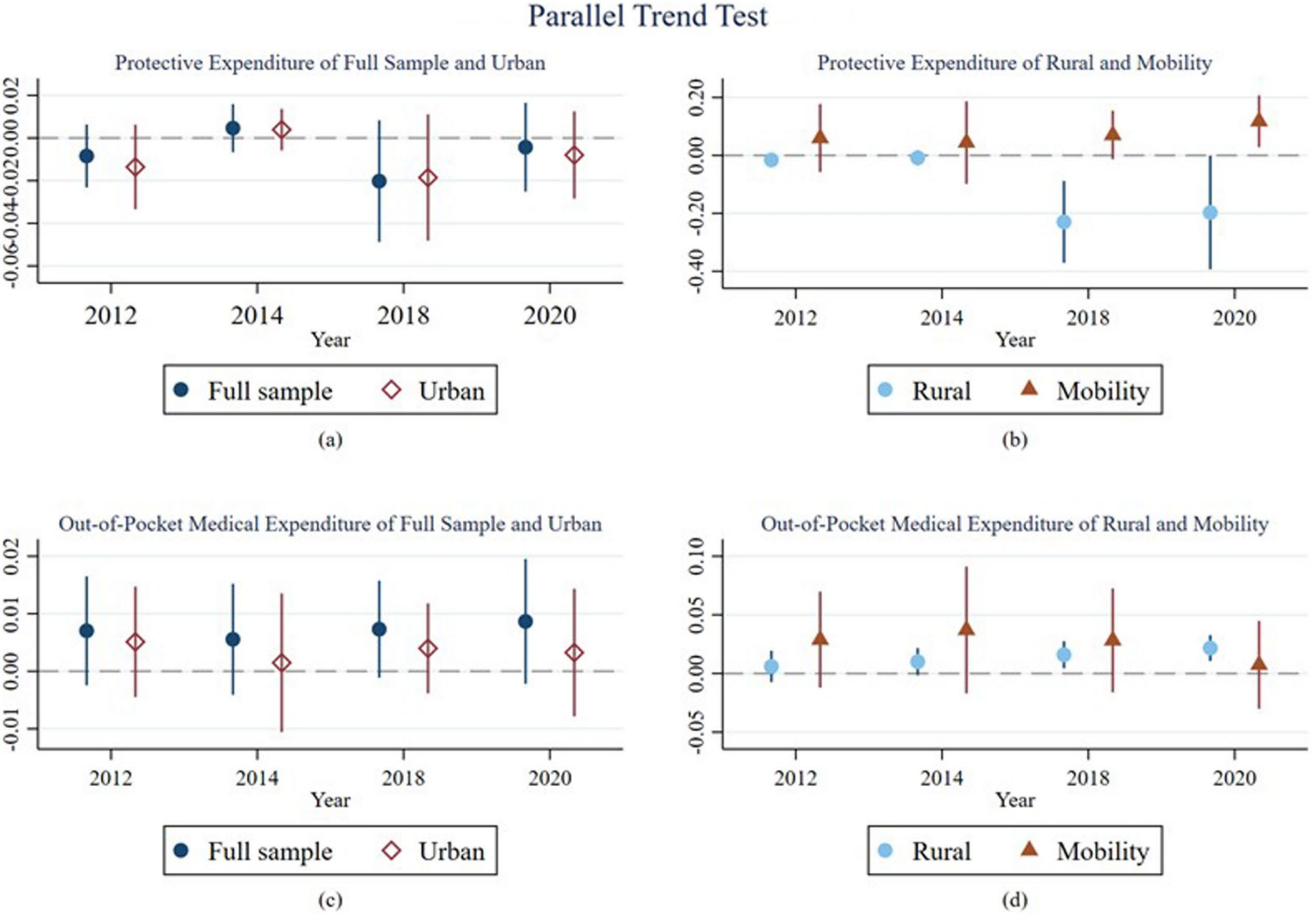
Parallel Trends Test. Figure shows that the parallel trend assumption test results for all samples. Among them, **(A)** and **(B)** show the parallel trend test results for the proportion of social security expenditures, while **(C)** and **(D)** display the parallel trend test results for the proportion of out-of-pocket medical expenditures. The confidence interval is 5%.

Figure 5 shows that the parallel trend assumption test results for all samples, including urban rural mobility households. Among them, (a) and (b) show the parallel trend test results for the proportion of social security expenditures, while (c) and (d) display the parallel trend test results for the proportion of out-of-pocket medical expenditures. The results of the parallel trends all meet the pre-assumed parallel trend assumption. There is a significant change at the 5% level in the protective investments and out-of-pocket medical expenditures for rural households, particularly a noticeable decrease in protective investments after the implementation of the NAMR. There is also a category of households that changed residence during the sample interval, which we call mobile households. This group of households experienced a significant increase in protected investments and a downward trend in out-of-pocket payments after the NAMR, although this result is not statistically significant.

#### 4.2.3 Sensitivity analysis

Due to the significant changes in the health expenditure behavior of rural households after the NAMR, this part conducts a sensitivity analysis of the baseline results of the rural sample from various perspectives. As shown in Table 5, columns (1) to (4) conduct counterfactual tests on the baseline results. In columns (1) and (2), the measure of household financial market participation is replaced with the proportion of household cash and deposits, and the results indicate that the asset management regulations have no effect on this subgroup. In columns (3) and (4), the policy implementation time is shifted forward to 2016, and the results remain insignificant. The analysis recognizes that NAMR may affect financial assets as well as liabilities. Consequently, in columns (5) and (6), we replace the intensity variable with the proportions of financial assets and financial debts, respectively. The results remain statistically significant.

**Table 5:** Robustness tests.

Variables	(1)	(2)	(3)	(4)	(5)	(6)	(7)	(8)	(9)	(10)
	Treat replaced with cash holding		1 year in advance		Debt		Cluster to province		Add interaction fixed effects	
	Y1_2	Y2_2	Y1_2	Y2_2	y1_2	Y2_2	Y1_2	Y2_2	Y1_2	Y2_2
Did_cas	−0.058	−0.011								
	(0.133)	(0.017)								
Did_pre1			−0.003	0.009						
			(0.009)	(0.006)						
Did_debt					−0.205**	0.012***				
					(0.071)	(0.005)				
Did							−0.205***	0.013**	−0.217**	0.013**
							(0.077)	(0.005)	(0.086)	(0.005)
Observations	8, 020	9, 390	8, 020	9, 390	8, 020	9, 390	8, 020	9, 390	7, 988	9, 388
Control	Yes	Yes	Yes	Yes	Yes	Yes	Yes	Yes	Yes	Yes
Family FE	Yes	Yes	Yes	Yes	Yes	Yes	Yes	Yes	Yes	Yes
Year FE	Yes	Yes	Yes	Yes	Yes	Yes	Yes	Yes	Yes	Yes

Table reports the results of robustness checks. Columns (1), (3), (5), (7), and (9) present the robustness test results for rural households’ protective investment. Columns (2), (4), (6), (8) and (10) show the test results for rural households’ out-of-pocket medical expenditures, respectively. The standard errors from clustering to the county level are enclosed in brackets. Significance levels are indicated as follows: *10%, **5%, and ***1%.

#### 4.2.4 Other robustness test

Considering the inter-city influences on healthcare, we cluster the standard errors at the provincial level in columns (7) and (8). In columns (9) and (10), to address the potential missing variables such as local policies and natural disasters that may affect medical expenditures, we include province-year interaction fixed effects. The results remain robust.

### 4.3 Heterogeneity tests

The differential effects of the same financial policy across different samples are related to household existing capital. Previous literature has confirmed that both tangible and intangible capital influence household health decisions. Property is one of the most important tangible assets for Chinese households, and rising property prices may reduce necessary health expenditures (54). However, if a household owns net property, this could influence their insurance choices in China (55). Intangible capital, such as trust and social networks, promotes household insurance purchasing behavior (56–59). Additionally, household social interaction experiences, such as caregiving and claims, can affect medical expenditures and the purchase of health insurance (60, 61). Based on this, in the heterogeneity analysis, this study examines the factors influencing the urban–rural differential response to the NAMR from three aspects: household asset allocation, occupational environment, and social interactions.

Table 6 reports the results of this analysis. First, Panel A tests the moderating effect of property ownership on health expenditure behavior. The results show that urban households with more property are less likely to engage in the purchase of protective assets. Specifically, a one-standard-deviation increase in net property holdings, combined with a 10-percentage-point increase in treatment intensity, is associated with an additional 0.067% reduction in the share of health expenditure. This reflects the wealth substitution effect, where real estate investments may displace guaranteed asset allocations in response to the policy shock. Although the magnitude is modest, this finding suggests that real estate holdings may crowd out guaranteed asset allocations following the policy shock. This also implies the significant importance of this substitution effect for families in first-tier cities, where net property holdings often amount to several million yuan or more. Panel B shows that, across all samples, households with business experience are more likely to invest in protective assets when facing investment uncertainty. For households with business experience, a 10-percentage-point increase in treatment intensity is associated with an additional 5.4% increase in the share of health expenditure. This reflects the advantages of financial literacy and social capital, as entrepreneurial families are more adept at transforming the impact of financial asset adjustments into health investments, rather than simply cutting expenditures. Panel C reports the moderating effect of social interactions. The study measures the level of household social interactions using the proportion of communication and transportation expenditures. For every one-unit increase in transportation expenditure share, combined with a 10-percentage-point increase in treatment intensity, the share of health expenditure increases by an additional 5.8%. The results indicate that rural households with higher social interaction expenditures are more likely to purchase protective assets after the implementation of the NAMR.

**Table 6:** Heterogeneity tests.

Variables	(1)	(2)	(3)	(4)	(5)	(6)
	Y1	Y1_1	Y1_2	Y2	Y2_1	y2_2
Panel A: net real estate assets						
Interaction	−0.003	−0.006*	−0.001	−0.001	0.001	−0.069
	(0.004)	(0.004)	(0.026)	(0.002)	(0.002)	(0.071)
Panel B: engaged in business						
Interaction	0.514***	0.541*	0.609*	−0.121	−0.104	0.093
	(0.194)	(0.299)	(0.324)	(0.091)	(0.098)	(0.154)
Panel C: social interaction						
Interaction	0.162	0.135	0.583*	0.089	0.097	−0.036
	(0.117)	(0.125)	(0.345)	(0.080)	(0.114)	(0.061)
Control	Yes	Yes	Yes	Yes	Yes	Yes
Family FE	Yes	Yes	Yes	Yes	Yes	Yes
Year FE	Yes	Yes	Yes	Yes	Yes	Yes

Table presents the results of heterogeneity analysis. Panel A reports the moderating effect of net real estate assets. Considering the large magnitude of household net property and for improved readability of the results, we have changed the unit of household net property to millions Panel B reports the moderating role of household business experience. Panel C reports the moderating effect of social networks. Columns (1), (3), and (5) present the regression results for migrant households. Columns (2), (4), and (6) present the regression results for rural households. The standard errors from clustering to the county level are enclosed in brackets. Significance levels are indicated as follows: *10%, **5%, and ***1%.

The heterogeneity results provide potential explanations for the urban–rural differences observed in the baseline regression. When facing investment uncertainty, households with higher business participation and more frequent social interactions tend to allocate funds to other commercial sectors, such as protective investments. Rural households generally have less business experience and fewer social interactions, which may lead them to avoid shifting their funds to protective investments or other areas when the investment environment changes.

The lack of significant changes in medical expenditures for urban households after the implementation of the NAMR could also be due to the substitutive relationship between property ownership and protective investments. Urban households, holding increasingly valuable property, may substitute this asset for the need for additional protective investments.

### 4.4 Were urban households affected?

The impact of investment choices among urban households is further explored in this section. In the baseline regression, urban households’ medical expenditure was not significantly affected by the asset management regulations. Considering the complementary relationship between education and health investments, this section examines the effect of the asset management regulations on household education expenditure to further investigate the underlying mechanisms. The results in Table 7 show that, after the implementation of the asset management regulations, urban households significantly increased their investment in education, while no significant effect was observed for rural or migrant households. This finding further illustrates the differential impact of financial policy changes, with urban households demonstrating a greater tendency to reallocate funds toward education.

**Table 7:** The impact of NAMR on education spending.

Variables	(1)	(2)	(3)	(4)	(5)	(6)
	Y3	Y3	Y3_1	Y3_1	Y3_2	Y3_2
Did	0.007	0.009*	0.014**	0.014**	−0.018	−0.012
	(0.006)	(0.005)	(0.006)	(0.006)	(0.014)	(0.010)
Observations	14, 295	14, 295	5, 005	5, 005	6, 710	6, 710
Control	No	Yes	No	Yes	No	Yes
Family FE	Yes	Yes	Yes	Yes	Yes	Yes
Year FE	Yes	Yes	Yes	Yes	Yes	Yes

Table reports the impact of the new asset regulation on the share of education expenditure as a percentage of the share. Columns (1), (3), and (5) present the regression results for the full sample, urban sample, and rural sample, respectively. Columns (2), (4), and (6) present the regression results for the full sample, the urban sample, and the rural sample, respectively, after adding control variables. The standard errors from clustering to the county level are enclosed in brackets. Significance levels are indicated as follows: *10%, **5%, and **1%.

The preceding analysis primarily focuses on the investment decisions of urban and rural households following the implementation of the asset management regulations. However, an important category of households has been overlooked—those whose residence moved between urban and rural areas during the sample period, referred to as ‘migrant households. Compared to urban households, migrant households may have lower existing levels of security and protection; yet, relative to rural households, they may possess more extensive social networks and greater entrepreneurial experience. To address this gap, this study further investigates the investment choices of migrant households after the enactment of the asset management regulations. Table 8 presents the behavioral changes of migrant households post-regulation: Columns (1) to (3) report adjustments in guaranteed investments, out-of-pocket medical expenditures, and educational expenditures among migrant households. The results indicate that, in contrast to rural households, migrant households significantly increased their guaranteed investments following the regulations. Columns (4) to (9) reveal heterogeneity in how different migrant households responded to the policy. Consistent with the findings in Table 6, migrant households with entrepreneurial experience demonstrated a greater propensity to increase guaranteed investments. Detailed results of robustness checks are provided in the Appendix.

**Table 8:** The role of NAMR for migrant families.

Variables	(1)	(2)	(3)	(4)	(5)	(6)	(7)	(8)	(9)
	Y1_3	Y2_3	Y3_3	Net real estate assets		Engaged in business		Social interaction	
				Y1_3	Y2_3	Y1_3	Y2_3	Y1_3	Y2_3
Did	0.068***	−0.005	0.013						
	(0.022)	(0.022)	(0.016)						
Interaction				−0.037*	−0.013	0.661*	−0.396	0.999	−0.040
				(0.020)	(0.010)	(0.347)	(0.271)	(0.869)	(0.174)
Observations	2, 790	3, 155	2, 580	2, 790	3,155	2, 790	3,155	2, 790	3,155
Control	Yes	Yes	Yes	Yes	Yes	Yes	Yes	Yes	Yes
Family FE	Yes	Yes	Yes	Yes	Yes	Yes	Yes	Yes	Yes
Year FE	Yes	Yes	Yes	Yes	Yes	Yes	Yes	Yes	Yes

Table reports the impact of the new asset regulation on the share of education expenditures. Columns (1)–(3) show the changes in protection investments, out-of-pocket healthcare expenditures, and education expenditures of migrant households after the new asset regulation. Columns (4)–(9) report the variability in the response of different migrant households to this policy. The standard errors from clustering to the county level are enclosed in brackets. Significance levels are indicated as follows: *10%, **5%, and ***1%.

## 5 Discussion

### 5.1 Key findings

This study reveals the spillover effects of financial policies in the health domain, with a particular focus on the significant heterogeneity in health expenditures between urban and rural households following the implementation of the NAMR. Households of different urban–rural types exhibit differing health consumption behaviors when confronted with the same policy. These differences are primarily influenced by household capital, including both tangible and intangible assets.

Our study first reveals the spillover effects of financial policies in the health domain, particularly highlighting the urban–rural differences in these effects. The impact of the NAMR on households extends beyond financial investments, influencing their consumption decisions in the health sector. Furthermore, we found significant heterogeneity in health expenditures between urban and rural households following the NAMR, with rural households experiencing a notable decline in protective investments and an increase in out-of-pocket medical expenses, thereby confirming the poverty trap hypothesis in the rural household sample. In contrast, urban households showed no changes in their health expenditures, including both protective investments and out-of-pocket expenses. Migrant households exhibited a different response, increasing their protective investments, although this change was not statistically significant. The observed differences in health consumption behavior across urban and rural households in response to the same financial policy may be related to the existing capital of the households, including both tangible and intangible capital.

Secondly, this study examines the impact of both tangible and intangible capital on health investment. The results show that urban households with higher net property holdings have a lower preference for health protection investments, indicating that tangible assets, such as property, can to some extent substitute for health investments. Specifically, between 2012 and 2020, China’s real estate market experienced rapid growth, and urban properties were generally viewed as having long-term appreciation potential. As a result, urban households with higher net property holdings tend to allocate more of their funds to real estate and reduce their allocation to other protective assets. This suggests that, despite the increased liquidity of funds due to the NAMR, urban households, due to their existing high-quality asset reserves, do not significantly increase their investment in health protection. Additionally, intangible assets, such as business experience, enhance a household’s preference for protective investments after the NAMR. It is noteworthy that households with business experience are more prevalent among migrant households than rural households, which may explain why migrant households and rural households exhibit nearly opposite attitudes toward protective investments.

Thirdly, we found that social capital—an intangible asset—has a positive moderating effect on household health protection expenditures. Rural households with frequent social interactions often have more robust social support networks and better access to information, which makes them more cautious and rational in their health investment decisions. This enables them to more effectively utilize health protection and medical services. Through past experiences and social networks, they gain access to more resources and information, becoming more knowledgeable about health protection products, medical resources, and relevant support. Even in a flexible financial environment, these households with strong social connections can maintain stable health investments. Moreover, by leveraging their broader social support networks, they are able to reduce their economic burden and lower out-of-pocket medical expenses.

We further examine the changes in investment behavior among urban and migrant households following the implementation of the asset management regulations. The results reveal that urban households did not significantly increase their expenditures on healthcare but instead allocated more resources to education. Given that both education and healthcare are critical public service domains and exhibit a certain degree of substitutability, this finding suggests that urban households tend to prioritize increased investment in education when responding to the asset management regulations. Meanwhile, although migrant households show an upward trend in guaranteed investments, the increase has not yet reached statistical significance.

### 5.2 Policy implications

By distinguishing between prudential and active financial policies, we deepen the understanding of current research on the relationship between financial policies and household health expenditure behaviors in China in two dimensions. First, we systematically assess the mechanism and spillover effects of prudential financial policies on household health expenditure, filling the research gap in the assessment of cross-sectoral effects of policies, and revealing that financial policies are altered in mature market environments through adjustments in risk expectations and changes in liquidity conditions, which not only affect decision-making in the health sector but also trigger synergistic changes in cross-sectoral behaviors, such as household investment in education.

Secondly, based on the perspective of asset endowment heterogeneity, this study elucidates the differentiated responses of urban and rural households and mobile households to policy shocks. Urban households are able to transform policy shocks into investment in health capital by virtue of their highly liquid assets and financial literacy, while rural households are forced to cut back on health expenditures and fall into a negative cycle of ‘risk aversion-health deterioration-poverty exacerbation’ due to their reliance on low-liquidity intangibles (e.g., land contract rights) and policy sensitivity. The mobile households have a unique cross-domain resource allocation strategy.

Based on these findings, this study emphasizes the need for health policymakers to address the differential impacts of prudential policies on household health expenditures, particularly the disproportionate burden on rural families. The significant urban–rural disparity in responses to NAMR underscores the necessity for tailored financial interventions, as urban households may reallocate resources toward long-term investments like education while financially vulnerable rural families face reduced healthcare access.

To mitigate these inequities, we propose a dual intervention approach: (1) enhancing rural medical risk-pooling through healthcare vouchers to improve service utilization and offset policy impacts, and (2) developing an intelligent monitoring platform that integrates voucher distribution, insurance claims, and big data analytics through village-level apps to identify at-risk households and automate timely financial assistance, thereby strengthening rural health resilience. These measures aim to reduce growing health disparities during economic development while accounting for distinct urban and rural financial circumstances.

### 5.3 Limitations and future research direction

This study examines the impact of financial policies on health expenditure inequality in China, contributing to interdisciplinary research on healthcare financing and offering novel insights into fiscal policy design. However, several limitations should be acknowledged. First, due to data availability constraints, we lack detailed information on household financial assets, particularly the allocation of specific financial instruments (e.g., deposits, bonds, stocks, and funds) across different household types. This limitation somewhat restricts our analysis of the heterogeneous effects of New Asset Management Regulations (NAMR) on Chinese households’ financial portfolios. Second, while we primarily investigate the short-term effects of NAMR, the long-term implications warrant further exploration. Theoretically, NAMR’s reduction of market rigidities may enhance household participation in financial markets over time, potentially increasing long-term wealth accumulation and consequently improving health expenditure capacity. However, our current research design does not permit direct empirical testing of these long-term dynamics.

Moving forward, we anticipate that richer data on financial market participation in China will enable more precise identification of the mechanisms through which financial policies influence household healthcare expenditures. Additionally, we aim to expand research on the equity of household health spending by examining intra-household dynamics—such as intergenerational support, gender composition, and caregiving burdens—which may lead to unequal distribution of health resources among family members and, consequently, affect overall health expenditure equity. Finally, we intend to further develop our research framework by integrating fiscal policy analysis with demographic shifts. Specifically, we plan to investigate how population aging and declining fertility rates interact with fiscal policies to shape household health spending decisions.

## Data Availability

Publicly available datasets were analyzed in this study. This data can be found here: https://www.isss.pku.edu.cn/cfps/. After accessing the provided link, readers can navigate to the data request module located in the sidebar to apply for access to the data used in the article. The application process typically takes between 2 to 7 working days. Detailed instructions for the application procedure can be found at the following link: https://zhuanlan.zhihu.com/p/368387796. Further inquiries can be directed to the corresponding author.

## Footnotes

^1^Data from China Health Statistical Yearbook: https://data.cnki.net/yearBook/single?id=N2020020200&pinyinCode=YSIFE
