# Supplementary material for: The impact of prudent financial policies on the urban–rural household health expenditure disparity: evidence from China
Source: Front Public Health. 2025 May 27;13:1580812. doi: 10.3389/fpubh.2025.1580812 (PMC12148918; doi:10.3389/fpubh.2025.1580812)
Supplement: Supplementary file 1 [file Data_Sheet_1.docx]

**Appendix**

**1.Figure_A_1 presents the parallel trend test for urban household education expenditures.**


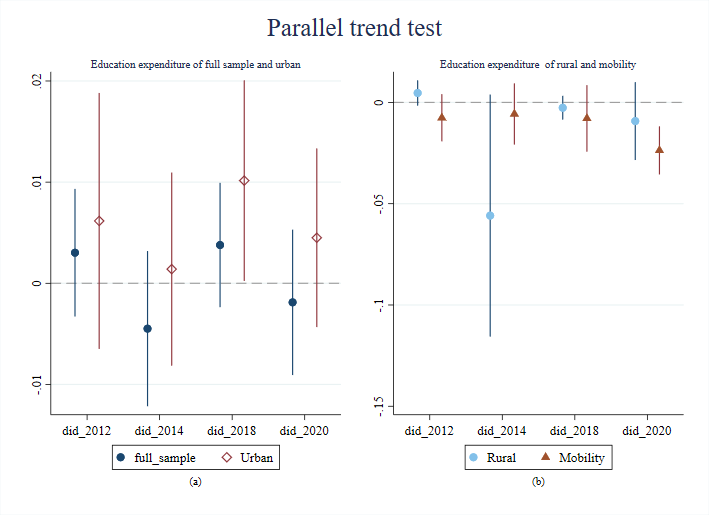


Note: The confidence interval is 5%.

**2.Table_A_1 the robustness test results for medical expenditures.**

|  | (1) | (2) | (3) | (4) | (5) | (6) |
| --- | --- | --- | --- | --- | --- | --- |
|  | y1 | y1_1 | y1_3 | y2 | y2_1 | y2_3 |
| Panel A：Treat replaced with cash holdings ratio | | | | | | |
| Did_cash | -0.002 | -0.006 | 0.125 | 0.006 | 0.006 | 0.014 |
|  | (0.011) | (0.010) | (0.094) | (0.005) | (0.005) | (0.026) |
| Panel B: 1 year in advance | | | | | | |
| Did_pre1 | -0.001 | 0.000 | 0.020 | -0.000 | -0.001 | -0.018 |
|  | (0.004) | (0.004) | (0.025) | (0.005) | (0.004) | (0.021) |
| Panel C：Cluster to province | | | | | | |
| Did | -0.008** | -0.009 | 0.068*** | 0.003 | 0.001 | -0.005 |
|  | (0.004) | (0.009) | (0.022) | (0.005) | (0.004) | (0.021) |
| Panel D: province-by-year interaction fixed effects | | | | | | |
| Did | -0.008** | -0.008 | 0.067** | 0.003 | 0.001 | -0.007 |
|  | (0.004) | (0.009) | (0.026) | (0.006) | (0.005) | (0.025) |
| Control | YES | YES | YES | YES | YES | YES |
| FE | YES | YES | YES | YES | YES | YES |
| Year FE | YES | YES | YES | YES | YES | YES |
